# Therapeutic Efficacy of Flavonoids in Allergies: A Systematic Review of Randomized Controlled Trials

**DOI:** 10.1155/2022/8191253

**Published:** 2022-04-13

**Authors:** Poliana Guiomar de Almeida Brasiel, Fernanda Verdini Guimarães, Patrícia Machado Rodrigues, Dumith Chequer Bou-Habib, Vinicius de Frias Carvalho

**Affiliations:** ^1^Laboratory of Inflammation, Oswaldo Cruz Institute, Oswaldo Cruz Foundation (FIOCRUZ), Rio de Janeiro, Brazil; ^2^Laboratory on Thymus Research, Oswaldo Cruz Institute, Oswaldo Cruz Foundation (FIOCRUZ), Rio de Janeiro, Brazil; ^3^National Institute of Science and Technology on Neuroimmunomodulation (INCT-NIM), Brazil

## Abstract

**Objective:**

To assess the clinical efficacy of flavonoid supplements on allergic diseases.

**Design:**

Systematic review. *Data Sources*. MEDLINE/PubMed, Scopus, Web of Science, and Embase databases were searched from inception to September 2021. *Eligibility Criteria for Selecting Studies*. Eligible study designs were randomized controlled trials that investigated the effect of flavonoids applied to allergic diseases.

**Results:**

This review included 15 randomized controlled trials, including allergic rhinitis/cedar pollinosis (*n* = 10), asthma (*n* = 3), and atopic dermatitis (*n* = 2). A total of 990 participants aged 6 to 69 years were included in these studies. Globally, 12 studies (80%) revealed some benefits of flavonoids (isolate or combined with other compounds) in allergic patients, while three studies (20%) reported no statistically significant impact on symptom scores and/or lung function. No severe adverse events related to treatment were reported. According to the GRADE system, the outcomes evaluated were of low to moderate quality of evidence.

**Conclusions:**

Overall, this review suggests that the administration of flavonoids may provide a viable strategy for mitigating allergic symptoms. Future trials with high methodological quality are needed to establish definitive conclusions. This trial is registered with PROSPERO registration no. CRD42021237403.

## 1. Introduction

Allergic diseases are a set of conditions caused by aberrant immunoglobulin (Ig) E-mediated reactions to allergen exposure. Many allergic diseases, including asthma, allergic rhinitis, allergic conjunctivitis, and atopic dermatitis, present multifactorial etiology and share similar risk factors [1, 2]. These diseases comprise an important cause of morbidity worldwide, causing a negative impact on the health and medical systems in both developed and emerging economies [3]. The estimated prevalence of allergic rhinitis was one in seven in U.S. adults and children (14% and 13%, respectively), 7% in Latin America, and 9% in the Asia-Pacific region [4]. According to the Global Asthma Report 2018, asthma affects as many as 339 million people worldwide and kills around 1,000 people every day [5]. Atopic dermatitis affects up to 20% of children and 10% of adults in high-income countries [6]. While there are efficient drugs widely used for the treatment of allergic diseases, conventional therapies do not often provide complete resolution of the symptoms and may present adverse effects associated with their continued use. Thus, new adjuvant therapies targeting the eliciting mechanisms of the allergic inflammatory response and able to improve the patient's quality of life are needed [7].

The development and progression of allergic diseases are related to the exacerbated inflammatory response [8]; thus, some foods and nutrients endowed with anti-inflammatory activity, such as flavonoids, can be useful in the management of these diseases [9, 10]. Flavonoids belong to a class of plant secondary metabolites widely found in fruits and vegetables [11]. Due to their antioxidant, anti-inflammatory, antiviral, and anticarcinogenic properties, flavonoids are components of a variety of nutraceutical, pharmaceutical, and cosmetic applications [12]. Preclinical and clinical reports have shown the efficacy and safety of flavonoids [13], although high doses of these compounds could potentially have adverse effects, including kidney or liver changes and anemia [14, 15].

Flavonoids, including flavones, flavonols, flavanones, isoflavones, and anthocyanins, may act on allergic diseases. Thus, patients with allergic disorders could benefit from flavonoid therapy alone or in combination with antiallergic drugs [16]. In this way, to contribute to a better evaluation of current clinical studies, we performed a critical analysis of randomized clinical trials investigating the effect of flavonoids on allergic diseases. We also aimed to improve the quality of the reports and to prevent the spreading of methodological failures, which could compromise the development of future studies and efficient clinical approaches.

## 2. Methods

This systematic review was elaborated according to the Preferred Reporting Items for Systematic Reviews and Meta-Analyses (PRISMA) [17, 18], whose methods include data source and search, study selection, eligibility criteria, data extraction, analysis of results, risk of bias, and quality of evidence. The protocol was registered at the International Prospective Register of Systematic Reviews (PROSPERO) (registration number: CRD42021237403).

### 2.1. Search Strategy

The bibliographic search was performed using the electronic databases MEDLINE (PubMed platform—https://www.ncbi.nlm.nih.gov/pubmed), Scopus (https://www.scopus.com), Embase (https://www.embase.com), and Web of Science (https://login.webofknowledge.com) to September 2021, admitting only randomized controlled trials. The keywords for the construction of the filters followed three criteria: allergy AND flavonoids AND randomized controlled trial (Supplementary file S1). The hierarchical distribution of the Medical Subject Headings (MESH) terms was the strategy used to develop the filter in the PubMed platform. We applied in the Scopus, Embase, and Web of Science platforms the same PubMed search strategy. Views, comments, notes, protocols, and unpublished studies were excluded.

No restrictions were imposed for language or date of publication. The bibliographies of the eligible studies were checked manually to find possible publications of interest. We also searched the site Clinicaltrials.gov for ongoing studies.

### 2.2. Selection of Studies

We included all the randomized controlled trials that evaluated the use of flavonoids either isolate or in combination with other compounds in allergic individuals—children and adults (population) compared to placebo (comparator). Prespecified eligibility and exclusion criteria were set using the PICOS (Population, Intervention, Comparison, Outcome, and Study design) strategy. The following exclusion criteria were used: (i) descriptive studies, such as annals of congresses, protocols, editorials, letters, case reports, and review works; (ii) animal studies; (iii) *in vitro* studies; and (iv) comparative and observational studies. Abstracts or unpublished reports were disregarded. Trials in which flavonoids were combined with prebiotics and/or probiotics were excluded due to their ability to alter the gut microbiota, influencing the immune system and the development of allergies [19, 20].

The evaluation of the eligibility of the studies was performed independently by two reviewers (P.G.A.B. and F.V.G.). In the case of disagreements, another group of reviewers (P.M.R.S., D.C.B-H., and V.F.C.) decided whether the study met the inclusion and exclusion criteria. Inclusion or exclusion was verified by evaluating the full text of potentially relevant studies.

### 2.3. Extraction and Synthesis of Data

A detailed examination of the studies was carried out to evaluate the strength of the evidence and the validity of their inclusion in this review. Data extraction and compilation tables were developed according to the following information: (i) publication characteristics such as authors, year, and country; (ii) features of the participants, including clinical manifestation/allergic disease, sex, age, and number of participants, besides main types of the intervention, like the type of flavonoids, dose, and duration of treatment; and (iii) effects of flavonoids and the main outcomes. When essential information was absent, the authors were contacted by us to get it.

Clinical evaluation (symptoms), lung function, atopy (skin prick tests), and immune biomarkers (total and specific IgE serum concentrations) were also considered. The asthma score, allergic rhinitis symptoms, and skin lesions were presented as primary outcomes. The data were subsequently compared, and conflicting information was identified and corrected after discussion among the reviewers.

### 2.4. Risk of Bias and Quality of Evidence

The risk of bias was analyzed using the Cochrane risk-of-bias tool for randomized trials (RoB 2.0) [21], updated versions for individually randomized parallel-group trial, and individually randomized crossover trial. The risk of bias was assessed in five distinct domains, with each answer leading to judgments of “low risk of bias,” “some concerns,” or “high risk of bias.” The Grading of Recommendations Assessment, Development, and Evaluation (GRADE) system was used to decide the quality of evidence for each outcome and generate an evidence profile table [22]. The GRADEpro GDT web version was used to assess the quality of the evidence [23].

## 3. Results

### 3.1. Study Selection

**Figure 1:**
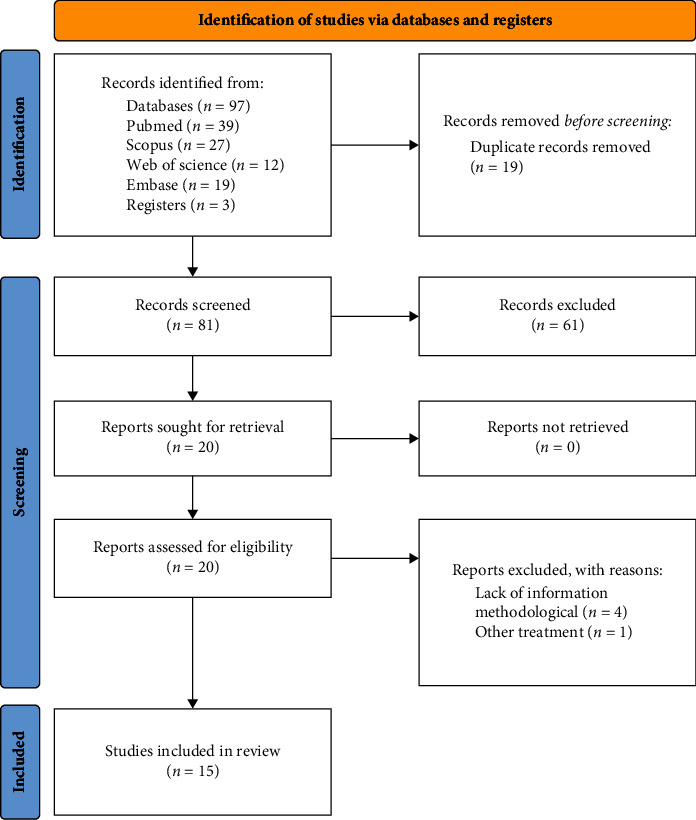
Flow diagram of the search results of our systematic literature review. Based on [18].

Our search strategy identified 93 bibliographic citations, of which 20 were selected for full-text assessment. No other additional study was identified in the gray literature search or through the screening of included studies' reference lists. We identified 15 randomized controlled trials comparing at least one flavonoid source with placebo and meeting the inclusion criteria. Of these, 5 were excluded for the following reasons: lack of methodological information (*n* = 4) and presence of other concomitant treatment (*n* = 1).  Figure 1 shows the flow diagram of the study selection process.

### 3.2. Study Characteristics

The selected 15 studies assessing the use of flavonoids in allergies were performed in 6 different countries, most of them in Japan (33.3%), followed by USA (20%), Iran (20%), Italy (13.3%), Canada (6.7%), and Thailand (6.7%). All studies were published in English. The articles investigated a variety of allergic conditions, including allergic rhinitis (*n* = 6), cedar pollinosis (*n* = 4), asthma (*n* = 3), and atopic dermatitis (*n* = 2). From 2004 to 2019, the 15 studies included a total of 990 participants, 498 in the intervention group and 476 in the control group. One trial is a crossover design with 16 subjects [24]. Individuals 6 to 69 years old were included in these studies. However, just two studies focused on the effects of flavonoids in allergic diseases of children and adolescents [25, 26]. In some trials, the age bracket has not been specified (*n* = 4, 26.6%), only the mean age. Regarding treatment duration, a variation of 3 days to 24 weeks was observed.

From these 15 articles, apple polyphenol was used in 2 trials [27, 28], and 2 studies were published by the same research group that assessed modified isoquercitrin, a quercetin glycoside [29, 30]. The commercial formulations Pycnogenol® [26, 31] and Lertal® [25], as well as several extracts such as from tomato [32], purple passion fruit [33], dodder seed [34], and shallot [35], were also evaluated. Soy isoflavone [36], silymarin [37], a combination of botanical products (Spanish needles (*Bidens pilosa*)), cinnamon (*Cinnamomum zeylanicum*), acerola (*Malpighia glabra*) [24], topical cream containing vitamin E, epigallocatechin gallate (EGCG), and grape seed procyanidins [38] complete the sources of flavonoids evaluated. The characteristics of all included studies are shown in Table 1.

### 3.3. Primary Outcomes

Flavonoids have been shown to be effective in the treatment of some allergic diseases, as demonstrated especially in symptom scores. Globally, 12 studies (80%) revealed some benefit of flavonoids (isolated or combined with other compounds) in allergic patients, while three studies (20%) reported no statistically significant impact in their findings. The symptom score was identified as a primary outcome; however, different scoring systems were employed. In general, the studies evaluated nasal symptoms, including itching, sneezing, rhinorrhea, and nasal congestion, and ocular symptoms such as itching, hyperemia of conjunctiva, and tearing. The main scale used was a 4-point scale based on the symptoms, in which 0 = absent or irrelevant, 1 = mild, 2 = moderate, and 3 = severe.

Apple polyphenols decreased nasal symptoms, such as sneezing, compared with placebo [27]. A high dose of apple polyphenols (200 mg) led to the improvement of sneezing attacks and nasal discharge, and a low dose (50 mg) reduced sneezing attacks and swelling of the nasal turbinate scores after 4 weeks of the intake [28]. Enzymatically modified isoquercitrin (100 mg/day) significantly improved total ocular symptoms during the whole period (8 weeks) [30], as well as the total ocular symptom plus medication score in patients with Japanese cedar pollinosis [29]. Improvement of the ocular symptom score was also observed in the patients who received oral shallot supplement (3 g/day) in combination with cetirizine [35]. Lertal®, an oral food supplement containing *Perilla frutescens* (80 mg), quercetin (150 mg), and vitamin D3 (200 IU), led to a significant reduction in allergic rhinoconjunctivitis exacerbation in children of ages ranging 6–12 years [25].

The Pycnogenol®, a standardized bark extract of the French maritime pine (*Pinus pinaster* Ait.), was evaluated in two trials—one with asthmatic patients and another with patients presenting allergic rhinitis. Pycnogenol treatment showed a positive effect on peak expiratory flow (PEF) and symptom scores compared to baseline in childhood asthma [26]. However, its use in individuals with allergic rhinitis was not able to significantly reduce total nasal and ocular symptom scores compared to placebo [31]. In addition, the purple passion fruit peel extract reduced the symptom score in asthmatic patients, including reduction in the prevalence of wheeze and shortness of breath, after 4 weeks of treatment, accompanied with an increase in the forced vital capacity (FVC) [33]. Following the investigation of the effects of flavonoids on asthma, soy isoflavone supplementation for 24 weeks did not significantly improve symptom scores (asthma symptoms utility index) and mean changes in prebronchodilator forced expiratory volume in the first second (FEV_1_) [36].

**Figure 2:**
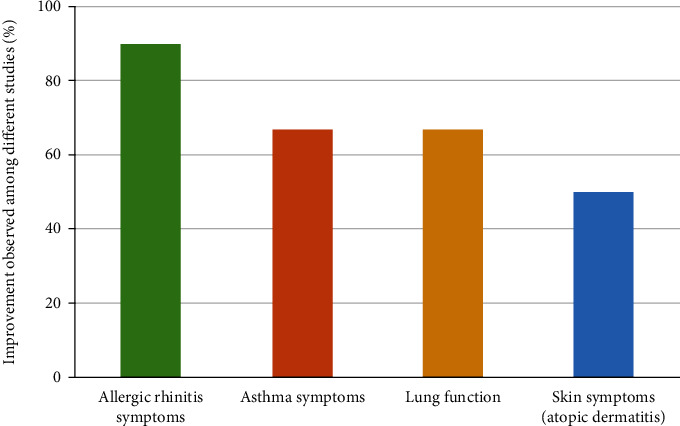
Percentage of improvement to each main outcome of the included studies.

In the case of atopic dermatitis, the treatment with whey associated with dodder seed extract improved skin moisture and elasticity compared to the placebo group [34]. Another work with atopic dermatitis showed no difference in the lesion skin area on the face and neck after topical cream containing vitamin E, epigallocatechin gallate (EGCG), and grape seed procyanidin treatment [38].  Figure 2 presents the percentage of improvement for each outcome of the included studies.

### 3.4. Quality of Life

Four reports assessed the degree of quality of life (QOL) [29, 30, 32, 36], two of them by using the Japanese allergic rhinitis QOL questionnaire (JRQLQ) [29, 30]. The JRQLQ comprises questions about rhinorrhea, sneezing, nasal obstruction, nasal and ocular itching, lacrimation, general fatigue, irritability, depression, and difficulties with daily activities such as working, housekeeping, studying, reading, doing sports, going outdoors, sleeping, and having conversations [39]. Only one study identified a significant improvement in patients' QOL in the treatment group (tomato extract) compared to the control [32].

### 3.5. Serum IgE and Cytokines

Only 4 trials presented results of serum IgE. Of these, three indicated no significant changes in total IgE [29, 32] and birch allergen IgE [31] during the study period. One trial showed a significant rise in mean IgE after treatment with silymarin [37]. Regarding cytokine levels (IL-4, IL-5, and IFN-*γ*), silymarin did not show any significant difference [37]. On the other hand, treatment with enzymatically modified isoquercitrin reduced serum levels of thymus and activation-regulated chemokine (TARC), however did not alter the levels of IL-4, IL-5, IL-12, IL-13, and IFN-*γ* [30].

### 3.6. Adverse Events

Information on adverse events was reported in 14 of the 15 studies. Five trials described side effects in evaluated individuals. The major adverse effects described were gastrointestinal symptoms [28, 34, 35], irritant contact dermatitis [38], dizziness [31, 35], fatigue [35], headache [31, 35], and menstrual symptoms [36], however without significant differences between the groups.

### 3.7. Study Quality and Risk of Bias

**Figure 3:**
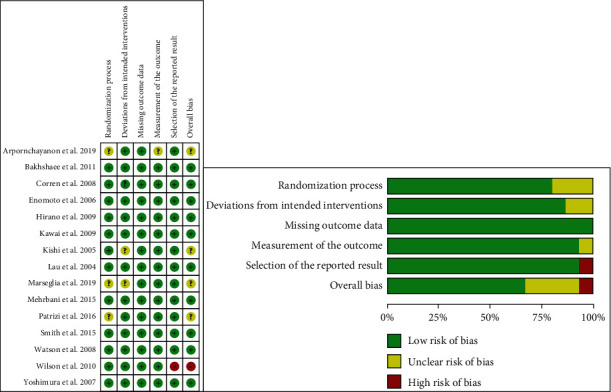
Results of risk of bias assessment were analyzed in all studies by revised Cochrane risk-of-bias tool for randomized trials (RoB 2).

Selected studies were evaluated using the revised Cochrane risk-of-bias tool for randomized trials (RoB 2) [21], as represented in Figure 3, considering the differences between parallel and crossover trials. An evidence profile according to GRADE for the included randomized trials is given in Table 2.

## 4. Discussion

We systematically reviewed the literature regarding flavonoid use in individuals with allergic diseases. We found 15 randomized controlled trials that evaluated at least 1 prespecified outcome, achieving a total of 990 participants. Although most studies had a low risk of bias, the quality of evidence was low to moderate grade given the small sample size and inconsistency of results. Despite limiting the strength of the present study's conclusions, several important findings appeared.

Allergic rhinitis, including pollinosis, was the allergic condition most assessed between the trials, with emphasis on Japanese studies. In particular, the number of patients with Japanese cedar pollinosis has increased [40], which seems to influence the largest number of studies conducted in this country. An important point observed is the lack of a standard symptom scale. Although Okuda's classification is widely used in Japanese studies, the application of different scales makes it difficult to compare different studies and summarize the results.

As observed in Table 1, different sources of flavonoids were evaluated, alone or in combination. Together, the results suggest that flavonoid use is beneficial in allergic diseases, such as allergic rhinitis, asthma, and atopic dermatitis, without causing serious adverse events. Nevertheless, the application of distinct interventions is a challenge in the compilation of studies for the same outcome. The flavonoids are widely found in dietary sources, including fruits, nuts, vegetables, and tea, with large variability depending on the cultivar, environmental factors, and preparation [14, 41]. The differences in bioavailability and absorption rates of diverse flavonoids are lacking in the included studies and need to be considered in the analysis of the results, because these pharmacokinetic parameters have an impact on health outcomes [42, 43]. These polyphenolic compounds are associated with a wide range of health benefits arising from their bioactive properties, such as anti-inflammatory, anticancer, antioxidant, cardiovascular benefit, neuroprotective, immunomodulatory, and antiviral properties [44]. These attributes drive flavonoids to medicinal use and development of functional products.

Due to immunomodulatory effects, several classes and sources of flavonoids were applied in subjects with allergic rhinitis/pollinosis, and 90% (9/10) of the studies identified some benefits associated with its use, such as the reduction of the symptoms. Nonetheless, the use of Pycnogenol® (100 mg/day), for 3 to 8 weeks prior to the birch pollen season, did not lead to significant improvement in nasal and ocular symptoms [31]. French maritime pine bark extract supplementation has already been studied under other pathophysiological conditions, as traumatic brain injury [45], urinary tract infections [46], osteoarthritis [47], among others [48] with different doses and duration, besides impact on clinical outcomes. The methodological limitations and imprecision lead to a low evidence level for this outcome, as can be seen in Table 2.

For asthma, a chronic airway inflammatory disease usually driven by the Th_2_ subset of CD4^+^ T lymphocytes [49], 67% (2/3) of the trials showed improvement in symptom scores and lung function. When evaluating the effects of soy isoflavone, no significant differences were observed in lung function, symptoms, and quality of life after 24 weeks of treatment. The inconsistency in results generated a moderate level of evidence for asthma symptoms and lung function. It is worth mentioning that soy isoflavones can act with endocrine disruption, and there is controversy regarding its consumption, especially during critical periods of development [50]. In addition, the low dosage and bioavailability of dietary supplements may have contributed to the lack of effects.

Two studies investigated the effects of flavonoids in atopic dermatitis. The combination of whey and dodder seed extract improved skin moisture and elasticity [34]. However, topical cream containing vitamin E, epigallocatechin gallate, and grape seed procyanidins showed no significant differences between the two treatment groups [38]. The small sample size and population heterogeneity make comparison difficult and lead to loss of evidence quality for this outcome.

The included studies employed a variety of extraction techniques and manufacturing practices, which makes it difficult to determine the possible impacts of these inconsistencies on the bioavailability and quality of the supplements evaluated. Another challenge in this review is the heterogeneity of outcomes reported across studies. Differences in age of participants (stage of life), source, dose, and duration of supplementation varied widely across the different studies, making it difficult to group the results and impacting the quality of evidence. It is worth pointing out that it is not possible to attribute the beneficial effects of flavonoid intake alone, but to a set of nutrients and bioactive compounds that are part of a healthy diet [51].

The methodological limitations and high heterogeneity of the studies included in the systematic review weaken the evidence about the real benefits of flavonoid intervention. Small sample sizes, limited numbers of randomized controlled trials per condition, variation in outcome measures, and incomplete reporting make it difficult to quantitatively compare studies and lead to the impossibility of settling definitive conclusions regarding the efficacy of flavonoid supplements for allergic diseases. Therefore, the findings of many of these trials need to be confirmed in larger and more rigorously designed clinical trials. The major strength of this work is that, to our knowledge, this is the first systematic review based on randomized controlled trials that summarize the efficacy of flavonoids on allergic diseases. Furthermore, in this study, we pointed out that the critical analysis following high methodological rigor can help to improve the quality of future reports and to assist in the clinical indication of flavonoid use in allergic diseases.

## 5. Conclusion

This systematic review found that different sources of flavonoids were associated with reduced symptoms in patients with allergic diseases. Although synthesized with low to moderate evidence quality, the findings suggested that flavonoid use is a viable strategy for mitigating allergic symptoms. Future research is needed to achieve the optimal dose, formulation, and duration of the treatment, as well as what is the best source of flavonoids. In this connection, more methodologically rigorous clinical trials are needed to generate quality clinical evidence about the use of flavonoids in the management of allergic diseases.

## Figures and Tables

**Table 1:** Baseline characteristics of the randomized controlled trials included in the systematic review.

Author, year	Country	Study design	Sample size (interv./placebo)	Sex (♂/♀)	Range age (mean years)	Allergic disease	Intervention	Dose/day	Study duration (wk)
Lau et al., 2004	USA	Parallel	28/26	NA	6-18 (14)	Asthma	Pycnogenol®	105 mg^∗^	13 wk
Kishi et al., 2005	Japan	Parallel	18/18	36/0	20-64 (NA)	Cedar pollinosis	Apple polyphenol	500 mg	12 wk
Enomoto et al., 2006	Japan	Parallel	22/11	9/24	15-65 (35)	Allergic rhinitis	Apple polyphenol	50 mg 200 mg	4 wk
Yoshimura et al., 2007	Japan	Parallel	17/16	14/19	18-56 (35)	Allergic rhinitis	Tomato extract	360 mg	8 wk
Watson et al., 2008	Iran	Parallel	22/21	16/27	18-60 (36)	Asthma	Purple passion fruit peel extract	150 mg	4 wk
Corren et al., 2008	USA	Crossover	16	NA	18-65 (38)	Allergic rhinitis	ClearGuard™	1350 mg	3 d
Kawai et al., 2009	Japan	Parallel	10/10	16/4	NA (39)	Cedar pollinosis	Enzymatically modified isoquercitrin	100 mg	8 wk
Hirano et al., 2009	Japan	Parallel	12/12	19/5	NA (36)	Cedar pollinosis	Enzymatically modified isoquercitrin	100 mg	8 wk
Wilson et al., 2010	Canada	Parallel	30/30	21/39	18-65 (44)	Allergic rhinitis	Pycnogenol®	100 mg	3-8 wk
Bakhshaee et al., 2011	Iran	Parallel	30/30	23/37	NA (29)	Allergic rhinitis	Silymarin	420 mg	4.3 wk
Smith et al., 2015	USA	Parallel	193/193	132/254	17-49 (34)	Asthma	Soy isoflavone	100 mg	24 wk
Mehrbani et al., 2015	Iran	Parallel	24/18	6/36	NA (28)	Atopic dermatitis	Whey+dodder seed extract	2000 mg	4.28 wk
Patrizi et al., 2016	Italy	Parallel	20/19	13/26	6-69 (27)	Atopic dermatitis	Vitamin E, EGCG, and grape seed procyanidins (MD2011001)	NA	4 wk
Marseglia et al., 2019	Italy	Parallel	64/64	NA	6-12 (9)	Allergic rhinitis	Lertal®	150 mg^∗∗^	4-12 wk
Arpornchayanon et al., 2019	Thailand	Parallel	8/8	5/11	18-65 (43)	Allergic rhinitis	Shallot extract	3 g	4 wk

Abbreviations: wk: week; d: day; NA: not available or unclear; USA: United States of America; ♂: male; ♀: female; PEF: peak expiratory flow; Pycnogenol®: mixture of water-soluble bioflavonoids extracted from the bark of French maritime pine (*Pinus pinaster* Aiton); ClearGuard™ combination botanical product: cinnamon (*Cinnamomum zeylanicum*) bark extract, acerola (*Malpighia glabra*) fruit concentrate, and Spanish needles (*Bidens pilosa*); EGCG: epigallocatechin gallate; Lertal®: quercetin 150 mg^∗∗^, *Perilla frutescens* 80 mg (as dry extract of the seeds containing rosmarinic acid, luteolin, apigenin, and chrysoeriol), and vitamin D3 5 mcg (200 IU). ^∗^Dose considering the average weight of a teen (47.6 kg).

**Table 2:** GRADE evidence profile: flavonoids for individuals with allergic diseases.

Quality assessment								
No. of studies (design)	Limitations	Inconsistency	Indirectness	Imprecision	Publication bias	No. of patients		Quality (GRADE)
						Flavonoids	Placebo	
Allergic rhinitis symptoms								
10 RCT	Serious limitations^a^	No serious inconsistency	No serious indirectness	Serious imprecision^b^	Undetected	214	202	⨁⨁◯◯ Low
Asthma symptoms								
3 RCT	No serious limitations	Serious inconsistency^c^	No serious indirectness	No serious imprecision	Undetected	243	240	⨁⨁⨁◯ Moderate
Lung function								
3 RCT	No serious limitations	Serious inconsistency^c^	No serious indirectness	No serious imprecision	Undetected	243	240	⨁⨁⨁◯ Moderate
Skin symptoms (atopic dermatitis)								
2 RCT	Serious limitations^d^	Serious inconsistency^c^	No serious indirectness	No serious imprecision	Undetected	44	37	⨁⨁◯◯ Low
Quality of life								
4 RCT	No serious limitations	Serious inconsistency^c^	No serious indirectness	No serious imprecision	Undetected	232	231	⨁⨁⨁◯ Moderate
Serious adverse events								
No studies reported this outcome.								

Abbreviations: GRADE: Grading of Recommendations Assessment, Development, and Evaluation; RCT: randomized controlled trials. ^a^Three trials have some concerns for risk of bias, and one study is judged to be at high risk of bias in one domain for this result. ^b^Different populations evaluated, follow-up time, and inconsistency in results. ^c^Important inconsistency (heterogeneity) in the results. ^d^One study is judged to be at some concerns for risk of bias.

## Supplementary Materials

Supplementary Materials Table S1: search strategy used on the PubMed, Scopus, Web of Science, and Embase databases.
