# Supplementary material for: Therapeutic Efficacy of Flavonoids in Allergies: A Systematic Review of Randomized Controlled Trials
Source: J Immunol Res. 2022 Apr 13;2022:8191253. doi: 10.1155/2022/8191253 (PMC9020920; doi:10.1155/2022/8191253)
Supplement: Supplementary Materials — Table S1: search strategy used on the PubMed, Scopus, Web of Science, and Embase databases. [file 8191253.f1.pdf]

Table 1S. Search strategy carried out on PubMed, Scopus, Web of Science and Embase databases.

DATABASE	DESCRIPTOR	RESULTS	DATA/TIME
PubMed	("hypersensitivity"[MeSH Terms] OR "hypersensitivity"[TIAB] OR "allergy and immunology"[MeSH Terms] OR "allergy and immunology"[TIAB] OR "allergy"[TIAB]) AND ("flavonoids"[MeSH Terms] OR "flavonoids"[TIAB] OR "quercetin"[MeSH Terms] OR "quercetin"[TIAB]) AND ("clinical trial"[MeSH Terms] OR "clinical trial"[TIAB] OR "controlled clinical trial"[MeSH Terms] OR "controlled clinical trial"[TIAB] OR "multicenter study"[MeSH Terms] OR "multicenter study"[TIAB] OR "randomized controlled trial"[MeSH Terms] OR "randomized controlled trial"[TIAB] OR "randomized controlled trials as topic"[MeSH Terms] OR "randomized controlled trials as topic"[TIAB] OR "double-blind method"[MeSH Terms] OR "double-blind method"[TIAB])	39	09/01/2021<br>20h15
Scopus	TITLE-ABS-KEY ((hypersensitivity OR allergy AND immunology) AND (flavonoids OR quercetin)) AND (LIMIT-TO (DOCTYPE , "ar")) AND (EXCLUDE (EXACTKEYWORD , "Animals") OR EXCLUDE (EXACTKEYWORD , "Animal") OR EXCLUDE (EXACTKEYWORD , "Mouse") OR EXCLUDE (EXACTKEYWORD , "Mice"))	27	09/01/2021<br>19h44
Web of Science	TOPIC: (hypersensitivity OR (allergy and immunology) OR allergy) AND TOPIC: (flavonoids OR quercetin) AND TOPIC: (clinical trial OR controlled clinical trial OR multicenter study OR multirandomized controlled trial OR randomized controlled trials as topic OR double-blind method)<br>Refined by: DOCUMENT TYPES: (ARTICLE)	12	09/01/2021<br>23h50
Embase	(hypersensitivity OR (allergy AND immunology) OR allergy) AND (flavonoids OR quercetin) AND [randomized controlled trial]/lim	19	09/01/2021<br>20h16
